# A descriptive analysis of midwifery education, regulation and association in 73 countries: the baseline for a post-2015 pathway

**DOI:** 10.1186/s12960-016-0134-7

**Published:** 2016-06-08

**Authors:** Sofia Castro Lopes, Andrea Nove, Petra ten Hoope-Bender, Luc de Bernis, Martha Bokosi, Nester T. Moyo, Caroline S. E. Homer

**Affiliations:** ICS Integrare, calle Balmes, 30,3-1, 08007 Barcelona, Spain; Women’s Health and Development, Geneva, Switzerland; UNFPA, 11-13 Chemin des Anémones, 1219 Chatelaine, Switzerland; ICM, Laan van Meerdervoort 70, 2517 AN The Hague, The Netherlands; University of Technology Sydney, 15 Broadway, Ultimo, NSW 2007 Australia

**Keywords:** Midwives, Education, Regulation, Association, Professional development, Sustainable Development Goals

## Abstract

**Background:**

Education, regulation and association (ERA) are the supporting pillars of an enabling environment for midwives to provide quality care. This study explores these three pillars in the 73 low- and middle-income countries who participated in the State of the World’s Midwifery (SoWMy) 2014 report. It also examines the progress made since the previous report in 2011.

**Methods:**

A self-completion questionnaire collected quantitative and qualitative data on ERA characteristics and organisation in the 73 countries. The countries were grouped according to World Health Organization (WHO) regions. A descriptive analysis was conducted.

**Results:**

In 82% of the participating countries, the minimum education level requirement to start midwifery training was grade 12 or above. The average length of training was higher for direct-entry programmes at 3.1 years than for post-nursing/healthcare provider programmes at 1.9 years. The median number of supervised births that must be conducted before graduation was 33 (range 0 to 240). Fewer than half of the countries had legislation recognising midwifery as an independent profession. This legislation was particularly lacking in the Western Pacific and South-East Asia regions. In most (90%) of the participating countries, governments were reported to have a regulatory role, but some reported challenges to the role being performed effectively. Professional associations were widely available to midwives in all regions although not all were exclusive to midwives.

**Conclusions:**

Compared with the 2011 SoWMy report, there is evidence of increasing effort in low- and middle-income countries to improve midwifery education, to strengthen the profession and to follow international ERA standards and guidelines. However, not all elements are being implemented equally; some variability persists between and within regions. The education pillar showed more systematic improvement in the type of programme and length of training. The reinforcement of regulation through the development of legislation for midwifery, a recognised definition and the strengthening of midwives’ associations would benefit the development of other ERA elements and the profession generally.

## Background

Tackling poor outcomes in sexual, reproductive, maternal and newborn health (SRMNH) in the post-2015 era requires a new paradigm for health, based on universal access and provision of essential quality services to all women and babies. Universal access demands a well-planned, prepared and enabled midwifery workforce [1–4].

There is increasing recognition of the role of midwives in improving SRMNH outcomes and of the environment enablers that improve the quality of midwifery practice. Recent evidence positions midwives as being pivotal to effective SRMNH services [3, 5]. Educated, regulated and licensed midwives, under adequate management and supervision, can increase the quality of care and positively impact on maternal and perinatal outcomes [2, 3, 5–7]. Midwives, when enabled to work to their full scope of practice within a functional health system, can operate across the whole continuum of care, as they are able to provide care from the community to referral facilities and from health promotion and education [5] to more complex life-saving interventions. Midwives can therefore increase the chances that women will seek care at the most effective time and level [6]. Additionally, evidence shows that midwives, when integrated within multidisciplinary teams and where adequate referral mechanisms are available, can improve SRMNH outcomes [5].

Several international organisations are involved in the strengthening of the midwifery profession, in particular the International Confederation of Midwives (ICM), the World Health Organization (WHO) and the United Nations Population Fund (UNFPA). With the aim of achieving global goals for SRMNH, ICM has identified three pillars for midwifery practice and development: *education*, to provide a competent, qualified workforce; *regulation*, to set the scope of practice, and licensing and re-licensing requirements ensuring that midwives provide high-quality midwifery care; and *association*, that consists of ‘an organised body of persons engaged in a common professional practice, sharing information, career advancement objectives, in-service training, advocacy and other activities’ and supports the strengthening of the workforce. ICM guides governments and midwives’ associations to develop and support their workforce based on these three pillars, collectively known as ‘ERA’ [8–13].

The State of the World’s Midwifery (SoWMy) 2011 [14] and 2014 [2] reports provided evidence on these pillars and on the policy environment in which midwives need to operate to achieve their potential and improve health outcomes. The reality is that high-quality SRMNH care is not always available and/or accessible [3]. The 2014 report showed severe health worker shortages: only 42% of the world’s midwifery, medical and nursing personnel are available to women and newborns in the countries where 92% of all the world’s maternal and newborn deaths and stillbirths occur [2].

If education, regulation and association (ERA) are key components for the development of a midwifery workforce able to provide quality care and respond to existing and future SRMNH challenges, it is important to understand how these components vary around the world. Understanding the variations can assist countries or associations to gauge their progress against global standards and benchmarks and provide opportunities for improvements or changes to be made. This study aims to explore specific aspects of ERA and highlight regional differences. It also takes stock of progress since 2011.

## Methods

In 2014, the second SoWMy report was published. The report was coordinated by UNFPA, WHO and ICM with, as technical partner, ICS Integrare. A steering committee was established, including donors and other partners. The 75 low- and middle-income countries included in the ‘Countdown to 2015’ [15], an initiative to track progress on maternal and newborn mortality and stillbirth rates in high burden countries, were invited to contribute to the report, and 73 did so (see the Appendix). These included countries from all six regions defined by WHO; however, they cannot be considered to be a representative sample of countries within each of the different regions. Ethical approval was received from the University of Southampton Ethics Committee (reference no 7688) prior to the commencement of data collection.

SoWMy 2014 collected detailed information on the midwifery workforce and the environment in which it operates. This process used two strands: a self-completion questionnaire, which collected quantitative and qualitative data about the workforce, its education, regulation, professional associations, policy and planning frameworks, and progress since the previous report from 2011 [14], and, for some, a national workshop involving national stakeholders and experts, to identify barriers and solutions for effective coverage of SRMNH care taking into account the four dimensions of effective coverage (availability, accessibility, acceptability and quality) [16, 17]. In this paper, we focus solely on the data from the self-completion questionnaire, which countries could choose to complete in English, French or Spanish.

The complete SoWMy 2014 questionnaire consisted of seven modules: (1) SRMNH workforce, (2) education and early career, (3) regulation, (4) professional health associations, (5) policies, (6) health infrastructure and workforce planning models and (7) actions taken in midwifery services since 2011 when the previous report was published. The analysis reported in this paper is taken from responses to selected questions in modules 2, 3, 4 and 7 and looks at ERA in more detail than was possible in the published SoWMy reports.

At country level, UNFPA and WHO nominated a lead technical midwifery/SRMNH worker as a focal point for the data collection. The focal points liaised with the Ministry of Health, midwifery associations, education providers, regulators and other key stakeholders to assist with the completion of the survey [18] and validation of the required data. The questionnaire was filled in and submitted via an online platform. A help desk was provided to assist users during the process. Anonymity was assured for those who preferred not to be acknowledged in the final report.

A descriptive analysis of the quantitative data was conducted using Excel (Microsoft Office) and Stata. Cross-regional analysis was conducted based on the WHO regional classification: Sub-Saharan Africa (hereafter AFRO), Eastern Mediterranean (EMRO), South-East Asia (SEARO), Western Pacific (WPRO), Americas (PAHO) and Europe (EURO).

A total of 54 countries took part in both SoWMy 2011 and SoWMy 2014 (see the Appendix). For these countries, change over time is examined where valid comparison could be made. We also analysed open-ended questions which asked countries to report their own perceptions of what had changed in relation to ERA since 2011.

## Results

The 73 participating countries represented all six of the WHO regions: 41 (56%) from AFRO, 9 (12%) from EMRO, 6 (8%) from SEARO, 6 (8%) from WPRO, 6 (8%) from PAHO and 5 (7%) from EURO. The overall results are strongly influenced by the AFRO region which made up over half of the sample. As noted earlier, the regional results cannot be considered to be representative of the region as a whole—just of the Countdown countries within that region.

### Education

In 60 countries (82%), the minimum requirement to start midwifery training was reported to be grade 12 or above. This proportion was highest in the AFRO region (95%) and lowest in the EMRO and EURO regions (around 60%). Within regions, there was more diversity in the entry requirements in EMRO, SEARO and EURO (Table 1).

**Table 1:** Characteristics of midwifery education and training, by region

		Countdown countries in					
	All countries	Sub-Saharan Africa	Eastern Mediterranean	South-East Asia	Western Pacific	Americas	Europe
	(*n* = 73)	(*n* = 41)	(*n* = 9)	(*n* = 6)	(*n* = 6)	(*n* = 6)	(*n* = 5)
Education							
Entry	*n* (%)	*n* (%)	*n* (%)	*n* (%)	*n* (%)	*n* (%)	*n* (%)
Minimum high-school requirement to start training, *n* (%)							
Less than grade 10	3 (4)	0	1 (11)	0	1 (16)	0	1 (20)
Grade 10 and above	7 (10)	1 (2)	3 (33)	2 (33)	0	0	1 (20)
Grade 12 and above	60 (82)	39 (95)	5 (55)	4 (66)	4 (66)	5 (83)	3 (60)
Missing^a^	3 (4)	1 (2)	0	0	1 (16)	1 (16)	0
Type of entry programme, *n* (%)							
Direct-entry	63 (86)^b^	35 (85)^c^	9 (100)	6 (100)^c^	3 (50)	5 (83)^c^	5 (100)
Post-graduation	8 (11)	6 (15)	0	0	2 (33)	0	0
Missing^a^	2 (3)	0	0	0	1 (17)	1 (17)	0
Training							
Years of study required to qualify (mean ± SD)	2.9 ± 0.9	3.0 ± 0.7	2.8 ± 0.8	2.8 ± 0.8	1.9 ± 0.7	4.0 ± 1.0	3.6 ± 0.9
Years of study required to qualify by type of entry (mean ± SD)							
Direct-entry	3.1 ± 0.8	3.1 ± 0.6	2.8 ± 0.8	2.5 ± 0.8	2.2 ± 0.8	4.0 ± 1.0	3.6 ± 0.9
Post-graduation	1.8 ± 1.0	1.8 ± 1.2	0	0	1.5 ± 0.7	0	0
Years of study required to qualify (median)	3.0	3.0	3.0	3.0	2.0	4.0	3.0
Years of study required to qualify (range)	1.0–5.0	1.0–4.0	1.0–4.0	2.0–4.0	1.0–3.0	3.0–5.0	3.0–5.0
Standardised curriculum, *n* (%)	67 (92)	39 (91)	7 (78)	5 (83)	6 (100)	5 (83)	5 (100)
Curriculum update after 2008, *n* (%)	56 (77)	31 (72)	6 (67)	4 (67)	6 (100)	5 (83)	4 (80)
Curriculum update after 2011, *n* (%)	38 (52)	20 (47)	4 (44)	3 (50)	4 (67)	5 (83)	2 (40)
Supervision							
Minimum number of supervised births in curriculum (mean ± SD)	43 ± 38.1	52 ± 43.3	44 ± 30.6	24 ± 15.2	40 ± 34.6	42 ± 31.9	9 ± 6.5
Minimum number of supervised births in curriculum (median)	33	45	28	20	20	50	5
Minimum number of supervised births in curriculum (range)	0–240	0–240	20–100	10–50	20–100	0–80	5–20
Minimum number of supervised births in curriculum, *n* (%)							
None	2 (3)	1 (2)	0	0	0	1 (17)	0
1–20	22 (30)	7 (17)	2 (22)	4 (67)	3 (50)	1 (17)	5 (100)
21–30	8 (11)	5 (12)	3 (33)	0	0	0	0
31–40	6 (8)	5 (12)	0	0	1 (17)	0	0
41–50	10 (14)	7 (17)	1 (33)	1 (17)	0	1 (17)	0
Above 50	16 (22)	11 (27)	2 (22)	0	1 (17)	2 (33)	0
Missing	9 (12)	5 (12)	1 (11)	1(17)	1 (17)	1 (17)	0

Note that the data only relate to the Countdown countries within each region and are not necessarily typical of the entire region

^a^Includes no reply and not applicable

^b^Three countries reported both direct-entry and post-graduation programmes.

^c^One country reported both direct-entry and post-graduation programmes

Direct-entry programmes for midwife education were reported by 63 countries (86%). Six AFRO and two WPRO countries reported post-graduation entry (usually after a nursing qualification), and three countries (one each in AFRO, SEARO and PAHO) reported both direct and post-graduation entry programmes. The mean duration of training was longer for direct-entry programmes at 3.1 years than for post-graduation programmes at 1.8 years. For direct-entry programmes, WPRO had the shortest average length of training (2.2 years), and PAHO, EURO and AFRO the longest (4.0, 3.6 and 3.1 years, respectively). Only countries in the WPRO and AFRO regions reported post-nursing programmes, and these were similar in terms of mean duration of education, 1.5 and 1.8 years, respectively.

Overall, 81% of countries met the ICM minimum standard of 3 years for a direct-entry midwifery education programme or 18 months for a post-graduate training. For direct-entry programmes, just 30% of WPRO countries met this standard, compared with 90% in the AFRO region. In post-graduate programmes, there was little variation between regions.

Nearly all countries (92%) reported having a standardised curriculum; however, only 52% had updated the curriculum in the preceding 3 years. Across regions, this ranged from 44% in the EMRO region to 83% in the PAHO region (Table 1).

Midwifery students need to provide care during labour and attend births under supervision [8]. The reported minimum number of supervised births to be completed before graduation ranged from 0 to 240 with a median of 33 (Table 1). There were 32 countries (44%) who reported 30 or more births, 2 reported no minimum at all and 9 did not report this information. The PAHO region had the highest median (50 supervised births), although this ranged from 0 to 80, and the EURO region the lowest median (5), because 3 out of 5 countries reported 5 although the responses ranged from 5 to 20.

The most frequently reported challenges to the provision of high-quality midwifery education were the recruitment of teaching staff (56 of the 73 countries or 77%), limited opportunities for students to obtain practical skills (*n* = 56, 77%) and the lack of or poor equipment available (*n* = 56, 77%). Alignment of the curriculum with midwifery competencies was the least mentioned challenge (*n* = 18, 25%). In the PAHO region, the unpreparedness of students from secondary education was also often reported. The WPRO region was particularly likely to report limited classroom space, as well as retaining staff capacity and the lack of opportunities for teachers to maintain and develop new skills (Table 2).

**Table 2:** Key challenges faced in the provision of quality education, by region

		Countdown countries in					
	All countries	Sub-Saharan Africa	Eastern Mediterranean	South-East Asia	Western Pacific	Americas	Europe
	(*n* = 73)	(*n* = 41)	(*n* = 9)	(*n* = 6)	(*n* = 6)	(*n* = 6)	(*n* = 5)
Education	*n* (%)	*n* (%)	*n* (%)	*n* (%)	*n* (%)	*n* (%)	*n* (%)
ᅟRecruit teaching staff	56 (77)	37 (90)	4 (44)	3 (50)	4 (67)	4 (67)	4 (80)
ᅟEquipment inadequacy	56 (77)	35 (85)	7 (78)	4 (67)	4 (67)	3 (50)	3 (60)
ᅟLack of opportunities for students	56 (77)	31 (76)	7 (78)	5 (83)	5 (83)	4 (67)	4 (80)
ᅟRecruit-qualified teaching staff	53 (73)	35 (85)	6 (67)	2 (33)	5 (83)	3 (50)	2 (40)
ᅟTeaching staff not enabled to keep skills	48 (66)	31 (76)	5 (56)	4 (67)	4 (67)	3 (50)	1 (20)
ᅟRetain staff	46 (63)	31 (76)	4 (44)	2 (33)	4 (67)	2 (33)	3 (60)
ᅟClassroom space	36 (49.3)	24 (58.5)	4 (44.4)	2 (33.3)	4 (66.7)	1 (16.7)	1 (20.0)
ᅟSecondary education provides inadequate preparation	34 (46.6)	20 (48.8)	3 (33.3)	1 (16.7)	4 (66.7)	4 (66.7)	2 (40.0)
ᅟClass too large	28 (38.4)	21 (51.2)	2 (22.2)	1 (16.7)	2 (33.3)	1 (16.7)	1 (20.0)
ᅟCurricula not aligned with midwifery competencies	18 (24.6)	10 (24.3)	2 (22.2)	1 (16.7)	1 (16.7)	2 (33.3)	2 (40.0)

Note that the data only relate to the Countdown countries within each region and are not necessarily typical of the entire region

### Regulation

Fewer than half of the countries (48%) said they had legislation recognising midwifery as an autonomous regulated profession. This was most lacking in the WPRO region, where none of the six countries reported such legislation, and in the SEARO region, only one did so. Despite this, 78% of the countries reported having an officially recognised definition of a professional midwife (ranging from 50% in WPRO to 100% in EURO), and in 92% of the countries, it was reported that a government department or government-approved organisation assumed a regulatory role (Table 3). However, some countries qualified their response by noting that the regulatory body was not fully functional or needed support to fulfil its duties.

**Table 3:** Characteristics of midwifery regulation, by region

		Countdown countries in					
	All countries	Sub-Saharan Africa	Eastern Mediterranean	South-East Asia	Western Pacific	Americas	Europe
	(*n* = 73)	(*n* = 41)	(*n* = 9)	(*n* = 6)	(*n* = 6)	(*n* = 6)	(*n* = 5)
Regulation	*n* (%)	*n* (%)	*n* (%)	*n* (%)	*n* (%)	*n* (%)	*n* (%)
Legislation recognising midwifery as an autonomous profession	38 (52)	18 (44)	4 (44)	5 (83)	6 (100)	2 (33)	3 (60)
A recognised definition of a professional midwife exists	57 (78)	35 (85)	6 (67)	4 (67)	3 (50)	4 (67)	5 (100)
A government body regulates midwifery practice	67 (92)	38 (93)	8 (89)	6 (100)	5 (83)	5 (83)	5 (100)
A licence is required to practise midwifery	33 (45)	20 (49)	4 (44)	5 (83)	0	3 (50)	1 (20)
A live register of licensed midwives exists	37 (51)	23 (56)	5 (56)	4 (67)	2 (33)	1 (17)	2 (40)

Note that the data only relate to the Countdown countries within each region and are not necessarily typical of the entire region

Respondents reported that regulatory organisations were responsible for the accreditation of education providers, setting standards for education and assuring the quality of education in 51 (70%), 49 (67%) and 39 (53%) countries (Table 4). They also had a role in continuing professional development, assessing competency prior to registration and establishing the scope of midwifery practice. There was some regional variation. In the AFRO, WPRO, PAHO and EURO regions, regulatory responsibilities included accreditation of education providers as one of the main activities as well as setting standards for education. The EMRO and SEARO regions also listed continuing professional development as one of the main activities of the existing regulatory bodies. PAHO highlighted establishing the scope of midwifery practice, and both AFRO and WPRO included assessing competency prior to registration among the main roles.

**Table 4:** Main activities undertaken by regulatory organisations, by region

		Countdown countries in					
	All countries	Sub-Saharan Africa	Eastern Mediterranean	South-East Asia	Western Pacific	Americas	Europe
	(*n* = 73)	(*n* = 41)	(*n* = 9)	(*n* = 6)	(*n* = 6)	(*n* = 6)	(*n* = 5)
Regulation activities	*n* (%)	*n* (%)	*n* (%)	*n* (%)	*n* (%)	*n* (%)	*n* (%)
Continuing professional development	51 (70)	28 (68)	6 (67)	6 (100)	3 (50)	4 (67)	4 (80)
Accreditation of education providers	51 (70)	29 (71)	5 (56)	3 (50)	4 (67)	5 (83)	5 (100)
Assessing competency prior to registration	49 (67)	29 (71)	5 (56)	4 (67)	4 (67)	3 (50)	4 (80)
Setting standards for education	49 (67)	29 (71)	6 (67)	4 (67)	1 (17)	4 (67)	5 (100)
Establishing the scope of midwifery practice	44 (60)	28 (68)	5 (56)	2 (33)	3 (50)	5 (83)	1 (20)
Ensuring the quality of education	39 (53)	25 (61)	4 (44)	3 (50)	2 (33)	2 (33)	3 (60)

Note that the data only relate to the Countdown countries within each region and are not necessarily typical of the entire region

### Professional associations

Professional associations open to midwives were widely available in all regions although many were not exclusive to midwives. Three quarters (74%) of countries had specific midwives association (Table 5); this ranged from 40% in the EURO to 85% in the AFRO region.

**Table 5:** Characteristics of midwifery associations, by region

		Countdown countries in					
	All countries	Sub-Saharan Africa	Eastern Mediterranean	South-East Asia	Western Pacific	Americas	Europe
	(*n* = 73)	(*n* = 41)	(*n* = 9)	(*n* = 6)	(*n* = 6)	(*n* = 6)	(*n* = 5)
Association	*n* (%)	*n* (%)	*n* (%)	*n* (%)	*n* (%)	*n* (%)	*n* (%)
Professional association exists that midwives can join	72 (97)	41 (100)	9 (100)	6 (100)	6 (100)	6 (100)	4 (80)
Midwives-only association exists	54 (74)	34 (85)	7 (78)	4 (67)	4 (67)	3 (50)	2 (40)
Associations’ roles							
Continuing professional development	68 (93)	39 (95)	8 (89)	6 (100)	5 (83)	5 (83)	5 (100)
Advising members of quality standards	68 (93)	40 (98)	7 (78)	6 (100)	5 (83)	5 (83)	5 (100)
Advising the government on the most recent National MNH or health policy document	61 (84)	36 (88)	6 (67)	6 (100)	4 (67)	6 (100)	3 (60)
Advising or representing members accused of misconduct or incompetence	50 (67)	32 (78)	4 (44)	5 (83)	3 (50)	3 (50)	3 (60)
Negotiating work or salary issues with the government	46 (63)	31 (76)	2 (22)	4 (67)	2 (33)	5 (83)	2 (40)

Note that the data only relate to the Countdown countries within each region and are not necessarily typical of the entire region

The roles of these associations were often related to continuing professional development and advising members on quality standards (93%), as well as advising the government on the most recent national SRMNH policy (84%) (Table 5). Similar results were observed across regions, except that associations in AFRO and PAHO were most likely to have a role as a negotiator on work or salary issues with governments.

### Changes between SoWMy 2011 and SoWMy 2014

Of the 54 countries which participated in both studies, 34 were from the AFRO region, 7 from EMRO, 5 from SEARO, 4 from WPRO and 2 each from PAHO and EURO. Since a different questionnaire was used in the two SoWMy reports, not all questions were directly comparable, so it was not possible to analyse time trends for all components of ERA. However, an overall improvement was observed between 2011 and 2014 for the questions common to both years (Table 6).

**Table 6:** Comparison of ERA elements between 2011 and 2014, by region

			Countdown countries in											
	All countries		Sub-Saharan Africa		Eastern Mediterranean		South-East Asia		Western Pacific		Americas		Europe	
	(*n* = 54)		(*n* = 34)		(*n* = 7)		(*n* = 5)		(*n* = 4)		(*n* = 2)		(*n* = 2)	
Education	2011	2014	2011	2014	2011	2014	2011	2014	2011	2014	2011	2014	2011	2014
Type of entry	*n* (%)	*n* (%)	*n* (%)	*n* (%)	*n* (%)	*n* (%)	*n* (%)	*n* (%)	*n* (%)	*n* (%)	*n* (%)	*n* (%)	*n* (%)	*n* (%)
Direct-entry only	19 (35)	45 (83)	13 (38)	28 (82)	4 (57)	7 (100)	1 (20)	4 (80)	1 (25)	3 (75)	0	1 (50)	0	2 (100)
Combined only	7 (13)	NA	4 (12)	NA	0	NA	2 (40)	NA	0	NA	1 (50)	NA	0	NA
Post-graduate only	6 (11)	6 (11)	4 (12)	5 (15)	0	0	0	0	0	1 (25)	1 (50)	0	1 (50)	0
More than one type	22 (41)	3 (6)	13 (38)	1 (3)	3 (43)	0	2 (40)	1 (20)	3 (75)	0	0	1 (50)	1 (50)	0
Length (in years)														
Direct-entry (all)														
<3	11 (20)	6 (11)	4 (12)	1 (3)	5 (71)	2 (29)	2 (40)	1 (20)	0	2 (50)	0	0	0	0
≥3	23 (43)	38 (70)	20 (59)	26 (77)	1 (14)	5 (71)	0	3 (60)	1 (25)	1 (25)	0	1 (50)	1 (50)	2 (100)
Missing	3 (6)	10 (19)	1 (3)	7 (21)	1 (14)	0	0	1 (20)	1 (25)	1 (25)	0	1 (50)	0	0
Post-graduate (all)														
<1.5	10 (19)	4 (7)	5 (15)	3 (9)	3 (43)	0	0	0	2 (50)	1 (25)	0	0	0	0
≥1.5	10 (19)	2 (4)	7 (20)	2 (6)	0	0	1 (20)	0	1 (25)	0	0	0	1 (50)	0
Missing	0	0	0	0	4 (57)	0	4 (80)	0	1 (25)	0	2 (100)	0	1 (50)	0
Regulation, *n* (%)														
Legislation exists recognising midwifery as an autonomous profession	19 (35)	24 (44)	15 (44)	20 (59)	1 (14)	3 (43)	3 (60)	1 (20)	0	0	0	0	0	0
A recognised definition of a professional midwife exists	39 (72)	44 (82)	28 (82)	31 (91)	2 (15)	4 (57)	4 (80)	3 (60)	3 (75)	3 (75)	0	1 (50)	2 (100)	2 (100)
A government body regulates midwifery practice	39 (72)	51 (94)	27 (79)	32 (94)	4 (57)	6 (86)	3 (60)	5 (100)	2 (50)	4 (100)	1 (50)	2 (100)	2 (100)	2 (100)
Association, *n* (%)														
Professional association exists that midwives can join	51 (94)	54 (100)	34 (100)	34 (100)	5 (71)	7 (100)	5 (100)	5 (100)	3 (75)	4 (100)	2 (100)	2 (100)	2 (100)	2 (100)
Midwives-only association exists	31 (57)	45 (83)	22 (65)	30 (88)	3 (43)	7 (100)	3 (60)	4 (80)	2 (50)	3 (75)	0	1 (50)	1 (50)	0

Note that the data only relate to the Countdown countries within each region and are not necessarily typical of the entire region

In education, there were many more direct-entry (only) programmes in 2014 than in 2011; in 2011, 19 countries had such programmes, and in 2014, 45 did, with increases observed in all regions. This was accompanied by an increase in the average duration of training towards international standards of at least 3 years for direct-entry programmes and 1.5 years for post-nursing programmes.

In 2014, 48 of the 54 countries reported that they had made progress in education since 2011, mostly in terms of the level of education, i.e. midwifery training was scaled up to international standards:

The length of the training programme for midwives was adjusted to last 3 years … the training programmes … were reviewed to align with ICM-WHO skills.

Countries mentioned the creation or development of a diploma and/or Licence-Master-Doctorate (LMD) system and the review of the curricula to focus on emergency obstetric and neonatal care (EmONC) competencies, competencies/skills, and/or international guidelines. Improvements to structures to increase the availability and quality of education were mentioned, including supervision and development of internship systems:

The curriculum has been designed; an obstetric laboratory is under construction along with a training project for teachers and internship supervisors.

Regulation also showed improvements overall. Between 2011 and 2014, seven countries introduced legislation recognising midwifery as an autonomous profession (five in AFRO and two in EMRO). On the other hand, in WPRO, PAHO and EURO no such progress was made, and 2 SEARO countries appeared to have retracted the legislation that existed in 2011 (in fact, one of these reported not having legislation in 2014 because it was at that moment in the process of evolving from a decree/act to higher legislation (Law/Act) on health professions, in which midwifery is recognised as an independent profession). Likewise, six countries (three in AFRO, two in EMRO and one in PAHO) said that a recognised definition of a professional midwife had been introduced since 2011. The most striking improvement related to regulation by a government-approved body was that between 2011 and 2014, 12 countries across 5 regions changed their response to this question from ‘no’ to ‘yes’ (5 in AFRO, 2 in EMRO, 2 in SEARO, 2 in WPRO and 1 in PAHO).

When asked to specify improvements in regulation since 2011, only 30 out of the 54 countries gave a response. In most cases, this involved the creation of a national board or council or the review or development of midwives’ scope of practice, for example:

We developed a scope of practice for midwifery, reviewed the midwifery handbook to include ICM competencies and global innovations.

Some countries also mentioned the development of a registration system or the creation of a code of conduct or an official definition of the profession:

The midwives code of conduct has been available since 2013 in its revised version, taking into account the current realities of the profession and technological advancements, including the list of equipments and materials essential for MNH and the list of medicine that can be prescribed by midwives with essential ICM skills.

With respect to professional associations, great improvements were seen. In 2011, 51 of the 54 countries had an association that midwives could join, and by 2014, all the 54 did. Perhaps more importantly, the number of countries with an association specifically for midwives increased from 31 to 45, and only the EURO region showed no progress.

More than two thirds of the 54 countries (*n* = 37) reported improvements in association, e.g. the creation of associations independent of other professions and the benefits of becoming an ICM member. Partnerships, participation and involvement in discussions regarding MNH care were also mentioned, particularly in policy- and decision-making:

The midwives association is often solicited to participate in different activities organised by the Health Ministry, notably to plan the sector.

Some countries also reported improvements in terms of leadership capacity and decentralisation processes.

### Limitations

This study has some limitations, which need to be taken into account when interpreting the findings. Although countries from all the WHO regions were involved in the study, it only included those low- and middle-income countries (LMICs) that are part of the Countdown to 2015 initiative. These countries are not necessarily representative of their regions, and findings may not be generalisable to other countries in the region. The data were provided by UNFPA and WHO focal points in each country, after consultation with relevant stakeholders. The results were validated within the countries, but no external validation or triangulation of the responses was carried out, so some data quality issues may remain in countries with weak health management information systems. In addition, the education information provided tended not to include private sector schools, where these exist. Neither did it include much information about practical elements of midwifery education, offering only an overview of the issues affecting classroom-based education.

## Discussion

This study explored the components of ERA of midwives in 73 LMICs and compared them across regions. Overall, the study showed that many LMICs are working towards international standards established for ERA, which support and enable the development of midwifery as a profession. However, not all components are being implemented equally and some variability persists among these LMICs, both between and within regions.

Overall, all the three ERA pillars have evolved and improved in these LMICs since 2011. Education is considered the key for quality of care and better health outcomes, as is the process of developing and maintaining competencies [14]; therefore, a larger investment may have occurred in this area. In 2014, the aspects that showed greatest progress towards international standards for education were the entry requirements for enrolment into a midwifery course, duration of training and use of a standardised curriculum [8, 9]. In all countries, and particularly those which do not apply international standards on entry level and duration of training (Botswana, Kenya, Myanmar, Papua New Guinea, Sierra Leone, Solomon Islands, South Africa, Viet Nam, Zambia and Zimbabwe), it would be interesting to conduct rigorous evaluations of education quality to determine whether increased duration of education programmes translated to improved quality of graduates*.* Other issues should be considered, in particular the organisation of the training. The West Africa Health Organisation (WAHO) is working on harmonising curricula with a common year of training for nursing and midwifery: it would be of interest to see if the ICM competencies are fully implemented in such a configuration. In some countries it may be a consequence of the choice to include the training of midwives within the LMD system, in an attempt to align their training structure with that of other medical/clinical professions.

Another issue is related to the proportion and quality of practical compared to theoretical education. Apart from the need for investment in the development of skills and competencies of education facilitators, students require adequately resourced supervised clinical practice [2, 19]. Many LMICs from different regions mentioned this as one of the main barriers to education. Quality and also duration of practical training are key aspects: those have to be taken into account when establishing common training with other students or developing new curricula.

Robust evaluation of the quality of theoretical and practical midwifery education is necessary to validate country efforts and to improve the quality of education. WHO and UNFPA are currently supporting such evaluations in a number of countries, but countries’ own evaluations of midwifery education and schools’ accreditation systems would bring about quality improvements, particularly in LMICs such as those involved in this study. The number of births attended by students during their education can be a proxy for quality [2, 20], and in this study, inconsistencies were observed between countries and regions on this measure. The source of such a disparity could be the absence of a reference or benchmark for the minimum number of supervised births, as evaluation is a subjective process which relies mostly on supervisor observation and taking a competency-based approach rather than one based on minimum numbers [8].

Regulation is critical for the protection of service users and of midwives themselves. Midwives should not only know the boundaries of their authorised practice but also maintain their practice within these boundaries. Some progress was observed since 2011 in the availability of a regulatory body, which often was the government itself, and the existence of a recognised definition of a professional midwife. Further research on these countries could provide insight whether there was improvement in quality of and access to SRMNH care in comparison to those who reported no change to legislative or regulatory mechanisms.

Our findings showed an increase in the number of LMICs with professional associations that are open to midwives, particularly associations specifically for midwives. Associations are more and more recognised as an important mechanism for strengthening the profession [2, 14]. Evidence shows the importance of an enabling work environment [5], and professional associations can contribute to this by optimising the value of midwives and providing a link between policy and implementation [21].

Across this group of LMICs, ERA has not evolved uniformly even where overall progress was strong. Our results show a high level of uniformity in the AFRO and PAHO regions. Since 2011, the WPRO and SEARO regions showed less consistent progress, while AFRO and EMRO showed more significant improvements towards international standards. This discrepancy could be due to countries having departed from very different start points. Alternatively, it may be an indication of the impact of SoWMy 2011 as an advocacy tool for midwifery and the use of a more standardised and coordinated approach to ERA, particularly in the AFRO and EMRO regions.

The 2014 findings showed there was still some variability within regions in relation to education. Education standardisation, which should guide countries to high-quality and competency levels of the health workforce, could be undermined by the implementation of short-term strategies (or short cuts) in terms of planning due to the pressure of severe health workforce shortfalls and/or high unmet need for SRMNH care [22].

The observed improvements in the AFRO region are particularly interesting as it is the region where relatively poor maternal and newborn health outcomes are observed. Our findings may be reflecting recent investments made in midwifery and SRMNH through development aid within the framework of Millennium Development Goals (MDGs) 4 and 5 and in the overall strengthening of health systems/workforce [1, 23]. The progress observed since 2011 and towards international standards could therefore lead to future health gains. On the other hand, the slow progress observed in health indicators in the region could also be related to the existence and persistence of key challenges for quality education [14, 24] as well as wider issues which impact on health outcomes.

In 2014, LMICs in the PAHO region reported the highest proportion of countries with legislation, especially in comparison to WPRO, where legislation was non-existent in all the countries included in this study. This might go some way towards explaining why education standards showed more variability and less development when compared against international standards and other regions. This may result from the growing number of private schools and of donor scholarships in the WPRO region, in particular in the Islands [25] which could be improved by a clear definition, legislative support and accreditation systems that would enable standardisation of programmes [25].

Despite the promising improvements in regulation since 2011, this is still the pillar where most variability lies. Between 2011 and 2014, the existence of legislation as well as the recognised definition supporting the midwifery professionals seemed to be left behind in LMICs in at least three regions. According to ICM’s Global Standards for Midwifery Regulation, legislation identifies who is a midwife and who is not and describes the scope of midwifery practice [10]. Regulation is built upon this legislation, establishing the set of criteria and processes that supports midwives to work autonomously within their full scope of practice [10, 14]. If there is no legislation to support regulation activities, the recognition and scope of midwife practice can be limited and inefficient, even if the government is the main regulator [26]. There is a lack of clarity in many countries around the role and scope of practice of the midwife, and this flows into uncertainties in education and practice. Every country needs to advocate for such clarity in legislation. Further research must also be conducted into the impact of clarity around the role and scope of practice on improvements in maternal and newborn health outcomes.

In the post-2015 agenda, the midwifery workforce will only be able to respond fully to SRMNH needs and new challenges if ERA are strong. This study pointed to some lines of action which could help to overcome challenges and improve ERA across regions, including the following: (1) simultaneous and holistic improvements to ERA as strengthening one in isolation is unlikely to address overall quality; (2) assuring the development or strengthening of legislation that recognises midwifery and could support education and association; (3) integration of ERA elements in national mechanisms of planning and strategy such as national SRMNH or health workforce plans; (4) making the best use of existing structures (e.g. associations and councils) which could advocate for ERA, increasing the involvement of stakeholders and providing evidence; regional observatories could coordinate regional actions and facilitate cooperation between countries to increase standardisation; (5) implementation of regular monitoring and evaluation activities and accountability mechanisms, which could provide timely and valuable information about progress and sustainability of investing in ERA for midwifery workforce development; and (6) conducting further research on ERA to improve and increase adaptability of ICM standards, including a better understanding of the interdependency between elements.

## Conclusions

This study showed that despite positive effects from recent investments in the midwifery workforce, among LMICs inter-regional disparities exist in education, regulation and association (ERA), the three fundamental pillars that support and enable professional development. Of these three pillars, education showed the most systematic improvement, although not uniformly across regions. The reinforcement of regulation through the development of legislation for midwifery, a recognised definition, as well as the strengthening of midwives’ associations would benefit the development of other ERA elements and the profession generally. Further work is needed to develop ERA in all countries to international standards and to accompany this investment with substantial progress on midwifery workforce management, so that the full potential of midwifery care can be realised.

## Abbreviations

AFRO, Africa Regional Office (WHO), covering sub-Saharan Africa; EmONC, emergency obstetric and neonatal care; EMRO, Eastern Mediterranean Regional Office (WHO); ERA, education, regulation, association; EURO, Europe Regional Office (WHO); ICM, International Confederation of Midwives; LMD, Licence-Master-Doctorate; LMICs, low- and middle-income countries; MDG, Millennium Development Goal; PAHO, Pan American Health Organization (WHO Americas Regional Office); SEARO, South-East Asia Regional Office (WHO); SoWMy, State of the World’s Midwifery; SRMNH, sexual, reproductive, maternal and newborn health; UNAIDS, Joint United Nations Programme on HIV/AIDS; UNFPA, United Nations Population Fund; UNICEF, United Nations Children’s Fund; WAHO, West African Health Organisation; WHO, World Health Organization; WPRO, Western Pacific Regional Office (WHO)

## Appendix

**Table 7:** List of participating countries, by region

AFRO (*n* = 41)	SEARO (*n* = 6)	WPRO (*n* = 6)	PAHO (*n* = 6)	EMRO (*n* = 9)	EURO (*n* = 5)
Angola	Bangladesh^a^	Cambodia^a^	Bolivia^a^	Afghanistan^a^	Azerbaijan
Benin^a^	India^a^	China	Brazil	Djibouti^a^	Kyrgyzstan
Botswana^a^	Indonesia^a^	Lao PDR^a^	Guatemala	Egypt	Tajikistan^a^
Burkina Faso^a^	Korea DPR	Papua New Guinea^a^	Haiti^a^	Iraq	Turkmenistan
Burundi^a^	Myanmar^a^	Solomon Islands	Mexico	Morocco^a^	Uzbekistan^a^
Cameroon^a^	Nepal^a^	Viet Nam^a^	Peru	Pakistan^a^	
Central African Republic^a^				Somalia^a^	
Chad^a^				Sudan^a^	
Comoros^a^				Yemen^a^	
Congo					
Congo DR^a^					
Côte d’Ivoire^a^					
Eritrea					
Ethiopia^a^					
Gabon^a^					
Gambia^a^					
Ghana^a^					
Guinea^a^					
Guinea-Bissau^a^					
Kenya^a^					
Lesotho					
Liberia^a^					
Madagascar^a^					
Malawi^a^					
Mali^a^					
Mauritania^a^					
Mozambique^a^					
Niger^a^					
Nigeria^a^					
Rwanda^a^					
Sao Tome and Principe					
Senegal^a^					
Sierra Leone^a^					
South Africa^a^					
South Sudan					
Swaziland					
Tanzania^a^					
Togo^a^					
Uganda^a^					
Zambia^a^					
Zimbabwe^a^					

^a^Also participated in SoWMy 2011
